# Comparison of energy expenditure and substrate metabolism during overground and motorized treadmill running in Chinese middle-aged women

**DOI:** 10.1038/s41598-020-58791-0

**Published:** 2020-02-04

**Authors:** Shuo Li, Jing-jing Xue, Ping Hong, Chao Song, Zi-hong He

**Affiliations:** 1 School of Sport Science, Shanghai University of Sport, Shanghai, China; 2 China Institute of Sport Science, Beijing, China; 3 Beijing Dance Academy, Beijing, China; 4 Winter Sports Administrative Center, General Administration of Sport of China, Beijing, China; 5 College of Sports Science, Tianjin Normal University, Tianjin, China

**Keywords:** Biological techniques, Biophysical methods

## Abstract

The purpose of this study was to compare differences of energy expenditure and substrate metabolism between motorized-treadmill and overground running in three different velocities in Chinese middle-aged women. In total, 74 healthy middle-aged women (age, 48 ± 4 years; height, 159.4 ± 4.9 cm; weight, 58.6 ± 6.7 kg; and body-mass index (BMI), 23.1 ± 2.7 kg/m^2^) volunteered to participate in this study. Bioelectrical-impedance analysis was used to measure body composition. Energy expenditure, carbohydrates (CHO), and fat oxidation were calculated with indirect calorimetry during motorized-treadmill and overground running. Running speed from slow to fast was 7.0, 8.0, and 9.0 km/h. The duration of each velocity was 6 min, separated by 5–15 min rest. There was no significant difference in energy expenditure between overground and treadmill running at the speed of 7 km/h (8.10 ± 1.25 vs. 7.75 ± 1.13 kcal/min, p > 0.05). Energy expenditure of overground running at 8 and 9 km/h was higher than that of treadmill running (9.36 ± 1.40 vs. 8.54 ± 1.21 kcal/min; 10.33 ± 1.55 vs. 9.54 ± 1.36 kcal/min; both p < 0.01). Fat contribution to energy consumption was significantly higher during treadmill running than during overground running (both p* < *0.01) at speeds of 8 and 9 km/h. Overground running at high intensity incurred greater energy consumption than treadmill running did. However, results showed greater fat utilization during treadmill running than during overground running at high intensity. It is critical that these differences are taken into account when we prescribe training modes and intensities for middle-aged women.

## Introduction

Multiple studies showed that physical inactivity is a major risk factor for morbidity and mortality^1,2^. A series of studies published on the Lancet from 2012 to 2016 indicated that physical inactivity is a global epidemic, and we should increase physical activity levels to prevent it^3–5^. Energy expenditure and substrate metabolism are important elements when considering physical activities. Choosing optimal activity modes and intensities according to the characteristics of energy expenditure and substrate metabolism may help prescriptions for improving quality of life.

Overground and treadmill running are two types of widely available movement patterns since they do not require special exercise skills. Treadmill running is commonly used in daily life, and a large number of runners regularly train on motorized treadmills, but whether physiological demands in treadmill running can be a substitute for overground running is unclear. Bidder^6^ suggested that outdoor track running (tarmac, grass) demands greater energy expenditure compared with a motorized treadmill at a same level. Aubry^7^ also found that a higher rate of oxygen uptake was needed in overground running than in treadmill running. However, several studies found the opposite results. Some studies^8,9^ found that there was no significant difference in oxygen uptake between the two modes, while others^10,11^ found that the oxygen uptake of motorized-treadmill running was higher than that of overground running at the same speed. Concerning the differences in subjects, test conditions, and methods, there is no consensus in previous researches on whether the energy demands of treadmill and overground running are similar. Carbohydrates (CHO) and free fatty acids are two main fuel sources that are oxidized during exercise, the contributions of which are influenced by physical activity intensity^12^, duration^13^, and exercise modes^14^. It should also be clear what the CHO and fat oxidation characteristics in overground and treadmill running are.

The majority of studies on the energy consumption of overground and treadmill running have been performed in young adults or athletes^6,7,15^. There is much evidence suggesting that findings on young subjects may not apply to other populations^16–18^. Bartolomeu *et al*.^16^ found that metabolic variables (heart rate, blood lactate concentration, oxygen uptake, energy expenditure) were significantly lower for older women at maximal intensity. Identical outcomes were reported by Campbell^18^ when comparing both age groups in similar conditions. Important reasons for energy expenditure changes were changes in age-related lactate production, stroke volume, arteriovenous oxygen difference, maximum heart rate (HR_max_), and musculoskeletal changes^19–21^. Reports in middle-aged and older adults are limited^22,23^, and all of which are comparisons between overground and treadmill walking. To date, there are no data on the energy-expenditure comparison of overground and treadmill running in middle-aged and older adults.

Given the above, the primary purpose of the present study was to compare the energy expenditure and substrate metabolism of overground and motorized-treadmill running at three different speeds in middle-aged women. We hypothesized that energy consumption and substrate utilization would be different during treadmill and overground running at the same speed.

## Materials and Methods

### Subjects

In total, 74 healthy middle-aged women were enrolled in this study. The eligibility criteria were for subjects to be healthy middle-aged women between 40 and 55 years with no recent experience of dieting or losing weight. Subjects were excluded if they were taking medication with known significant metabolism effects or if they were diagnosed with cardiovascular, respiratory, digestive-system, metabolic, bone and joint, thyroid, blood-system, and urinary-system diseases, or any condition that limited mobility. Participant characteristics are presented in Table 1. Subjects were told to wear comfortable clothes and shoes for all tests, and were asked to abstain from strenuous exercise, caffeine, and alcohol the day before the tests. They were also asked to arrive at the laboratory in a fasted state to eliminate the thermic effect of food (TEF).

**Table 1:** Characteristics of Subjects (n = 74, mean ± SD).

	Anthropometrical data (Mean ± SD)
Age (years)	48 ± 4
Height (cm)	159.4 ± 4.9
Weight (kg)	58.6 ± 6.7
Body mass index (kg/m^2^)	23.1 ± 2.7
FFM (kg)	41.1 ± 3.2
Body fat (%)	28.9 ± 6.9
Bust circumstance (cm)	86.4 ± 5.9
Waist circumstance (cm)	74.9 ± 7.8
Hipline circumstance (cm)	92.3 ± 5.9
WHR	0.81 ± 0.06
SBP (mmHg)	111 ± 10
DBP (mmHg)	72 ± 9

FFM Fat Free Mass, WHR waist-to-hip ratio, SBP systolic blood pressure, DBP diastolic blood pressure.

All subjects were asked to sign informed consent prior to the study. All procedures in this study were in accordance with the guidelines in the Declaration of Helsinki and were approved by the China Institute of Sport Science Committee (ethical code: CISSIRD-201604).

### Anthropometric measurements

The same trained tester performed all anthropometric measurements on subjects, namely, height, weight, body composition, and chest, waist, and hip circumference. Height was measured with the Su Heng Health Scale (RGZ-120, China). Weight and body-fat content was measured with a Body Composition Analyzer (INBODY 770, South Korea). Chest, waist, and hip circumference were measured by tape (SECA, Hamburg, Germany). We used the BMI = weight (kg)/height (m) squared formula to estimate the subject BMI.

### Energy-expenditure tests

Energy expenditure was measured by using indirect calorimetry (Metamax 3B-R2 metabolic measurement system, Germany). A standard mixture of known oxygen and carbon dioxide gas concentrations was used to calibrate the portable gas metabolism system to ensure precise sensor operation. A 3000 ml syringe was used to calibrate flow-sensor calibration. The idea behind the equipment is to use the method of every breath measurement to acquire real-time data of expired ventilation (VE), respiratory rhythm, oxygen consumption (VO_2_), carbon dioxide production (VCO_2_), and other parameters in the process of locomotion^24^. Room temperature and humidity were controlled to 22–25 °C and 40%–50%, respectively.

The energy-expenditure tests of treadmill and overground running were completed at an indoor gymnasium in Beijing. Before tests, the subjects were allowed to walk or run overground and on the treadmill for about 6 min to familiarize themselves with the test environment and conditions^25^, including running overground with a metronome and according to voice prompts (“fast, slow”, etc). A rest break of about 5 min was given to allow participants’ heart rates to recover to a level ± 5% of their resting heart rates^26^. After habituation with the test conditions, formal data collection was conducted. Treadmill and overground running speeds from low to high were 7, 8, and 9 km/h (commonly used speeds in regular populations). The duration of each speed level was 6 min whatever the running mode. The overground running field was a rectangular field with a 40 m perimeter (15 m long and 5 m wide). Pylons were placed every 5 m on the track to control subjects’ actual running speed. A sound signal was produced every 2.6, 2.2, and 2 s at 7, 8, and 9 km/h, respectively, and subjects had to adjust their speed to reach a pylon every time they heard a sound signal. A metronome to control speed was widely used by several studies^27–30^. Furthermore, every subject was accompanied by a pacesetter of our research team during their overground running. Treadmill running (Rodby RL3500E, Sweden) was completed in the same stadium to minimize environmental influences on performance. Stable-state data of the last 2 min of each speed level were used to calculate the energy consumption of overground and treadmill running. The definition of a steady state usually calls for 3–5 min where VO_2_ and VCO_2_ vary by <10%–15%^31,32^. Achievement of a steady-state period during exercise testing reduces error in the assessment of energy expenditure. The next speed level of the running test was started after the subject had recovered to a level ± 5% of their resting heart rates (at least 5 min)^26^. Subject heart rate was monitored during all running tests.

### Calculations

Energy expenditure was calculated using the following equation, assuming a negligible contribution of protein oxidation. Energy expenditure was calculated assuming that 1 g carbohydrate = 4 kcal, and 1 g fat = 9 kcal^33^. Fat and carbohydrate oxidation were calculated from respiratory measurements (VO_2_, VCO_2_) according to the table of nonprotein respiratory quotient^34^. CHO and fat oxidation contributions were calculated using the Dumortier formula^35^. All formulas are shown in Table 2.

**Table 2:** Formulas of energy expenditure and substrate metabolism.

Variables	Formulas
Energy expenditure (kcal/min)	fat oxidation (g/min) × 9 + carbohydrate oxidation (g/min) × 4
Fat oxidation (g/min)	1.695 × VO_2_ (l/min) − 1.701 × VCO_2_ (l/min)
Carbohydrate oxidation (g/min)	4.585 × VCO_2_ (l/min) − 3.226 × VO_2_ (l/min)
% Fat	((1 − RER) / 0.29) × 100
% CHO	((RER − 0.71) / 0.29) × 100

### Statistics

Two-way ANOVA 3 (speed: 7, 8, and 9 km/h) × 2 (mode: overground and treadmill running) with repeated measures was used to examine differences in energy expenditure, VO_2_, heart rate, CHO and fat oxidation, and fat contribution (%). Simple-effect analysis was applied to examine differences between treadmill and overground conditions. Results were analyzed using SPSS23.0. Significance level was set at p < 0.05. Effect size was evaluated with η^2^ (Eta partial squared), where 0.01 < η^2^ < 0.06 represented a small effect, 0.06 < η^2^ < 0.14 a medium effect, and η^2^ > 0.14 a large effect^36^.

## Results

**Figure 1:**
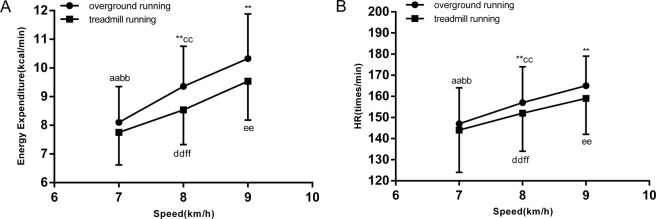
(**A**) Mean and standard deviations of energy expenditure for three different running speeds. (**B**) Mean and standard deviations of heart rate (HR) for three different running speeds. ^**^Compared with same speed on treadmill running p < 0.01; ^aa^p < 0.01 overground running of 7 vs. 8 km/h; ^bb^p < 0.01 overground running of 7 vs. 9 km/h; ^cc^p < 0.01 overground running of 8 vs. 9 km/h; ^dd^p < 0.01 treadmill running of 7 vs. 8 km/h; ^ee^p < 0.01 treadmill running of 7 vs. 9 km/h; ^ff^p < 0.01 treadmill running of 8 vs. 9 km/h.

**Figure 2:**
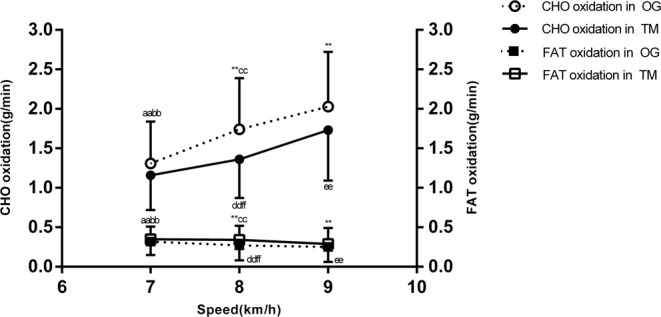
Mean of all participants’ carbohydrate (CHO) and fat oxidation for three different running speeds. ^**^Compared with same speed on treadmill running p < 0.01; ^aa^p < 0.01 overground running of 7 vs. 8 km/h; ^bb^p < 0.01 overground running 7 vs. 9 km/h; ^cc^p < 0.01 overground running 8 vs. 9 km/h; ^dd^p < 0.01 treadmill running 7 vs. 8 km/h; ^ee^p < 0.01 treadmill running of 7 vs. 9 km/h; ^ff^p < 0.01 treadmill running of 8 vs. 9 km/h.

**Figure 3:**
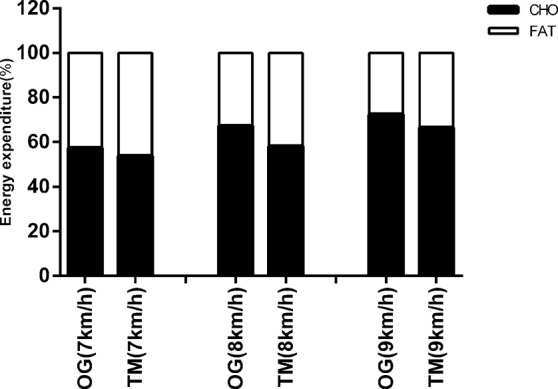
Mean values of contribution of CHO (%) and fat (%).

A significant effect of increasing the running speed from 7 to 9 km/h was found on energy expenditure (F = 229.7, p < 0.01, η^2^ = 0.76; Fig. 1A), VO_2_ (F = 294.3, p < 0.01, η^2^ = 0.80; Table 3), CHO oxidation (F = 119.4, p < 0.01, η^2^ = 0.62; Fig. 2), fat oxidation (F = 15.4, p < 0.01, η^2^ = 0.18; Fig. 2), fat contribution (%) (F = 63.3, p < 0.01, η^2^ = 0.47; Fig. 3), heart rate (F = 257.3, p < 0.01, η^2^ = 0.78; Fig. 1B). Simple-effect analysis showed that, with increasing running speed from 7 to 9 km/h on overground and treadmill running, energy expenditure, VO_2_, HR, CHO oxidation increased; however, fat oxidation and contribution (%) decreased.

**Table 3:** Energy expenditure and substrate metabolism during overground and treadmill running at same speed for middle-aged women (mean ± SD).

Speed	7(km/h)		8(km/h)		9(km/h)	
Variable	OG	TM	OG	TM	OG	TM
CHO oxidation (g/min)	1.31 ± 0.53^aabb^	1.16 ± 0.44^ddee^	1.74 ± 0.65^**cc^	1.36 ± 0.49^ff^	2.03 ± 0.69^**^	1.73 ± 0.64
Fat oxidation (g/min)	0.32 ± 0.17^aabb^	0.35 ± 0.16^ddee^	0.27 ± 0.19^**cc^	0.34 ± 0.18^ff^	0.25 ± 0.19^**^	0.29 ± 0.20
Contribution of fat (%)	42.68 ± 20.16^aabb^	46.13 ± 20.13^ddee^	32.62 ± 22.41^**cc^	41.98 ± 19.59^ff^	27.59 ± 20.38^**^	33.41 ± 21.32
EE (kcal/min)	8.10 ± 1.25^aabb^	7.75 ± 1.13^ddee^	9.36 ± 1.40^**cc^	8.54 ± 1.21^ff^	10.33 ± 1.55^**^	9.54 ± 1.36
VO_2_ (ml/min/kg)	27.36 ± 2.64^aabb^	26.77 ± 2.99^ddee^	30.83 ± 2.99^**cc^	29.28 ± 2.86^ff^	33.78 ± 3.59^**^	31.74 ± 2.87
HR (beats/min)	147 ± 17^aabb^	144 ± 20^ddee^	157 ± 17^**cc^	152 ± 18^ff^	165 ± 14^**^	159 ± 17

^**^p < 0.01 overground running vs. treadmill running with speed of 7, 8, and 9 km/h. ^aa^p < 0.01 overground running of 7 vs. 8 km/h. ^bb^p < 0.01 overground running of 7 vs. 9 km/h. ^cc^p < 0.01 overground running of 8 vs. 9 km/h. ^dd^p < 0.01 treadmill running of 7 vs. 8 km/h. ^ee^p < 0.01 treadmill running of 7 vs. 9 km/h. ^ff^p < 0.01 treadmill running of 8 vs. 9 km/h.

A significant effect of the modes was found on energy expenditure (F = 31.8, p < 0.01, η^2^ = 0.13; Fig. 1A), VO_2_ (F = 44.1, p < 0.01, η^2 ^ = 0.17, Table 3), CHO oxidation (F = 35.2, p < 0.01, η^2 ^ =0.14; Fig. 2), fat oxidation (F = 15.1, p < 0.01, η^2 ^ = 0.06; Fig. 2), fat contribution (%) (F = 38.9, p < 0.01, η^2^ = 0.15; Fig. 3), heart rate (F = 17.9, p < 0.01, η^2^ = 0.08; Fig. 1B). Simple-effect analysis showed that all variables during overground running at speeds of 8 and 9 km/h were higher than those during treadmill running at the same speed, but fat oxidation and contribution (%) were lower than those of treadmill running. However, there were no significant differences found between overground and treadmill running at 7 km/h (p > 0.05).

## Discussion

This study was aimed to compare the energy expenditure and substrate metabolism between overground and treadmill running for middle-aged women. The principal finding of the present study was that physiological variables (energy expenditure, oxygen uptake, heart rate) during overground running were significantly higher than those during motorized-treadmill running at speeds of 8 and 9 km/h. No variable was significantly different between overground and treadmill running at the speed of 7 km/h. As speed increased, the difference in VO_2_ and heart rate (HR) between overground and treadmill running increased. Moreover, regardless of overground and treadmill running, with increased exercise intensity, the relative contribution of fat oxidation to total energy expenditure decreased, whereas the contribution of carbohydrate oxidation increased.

Niemeyer^37^ compared the energy and carbohydrate demand for interval training on a track and treadmill, and found that the track demand was higher than that of the treadmill. Aubry^7^ also found metabolic demand on an outdoor track was significantly higher compared with that of a treadmill. Consistent with previous studies, our results showed that the energy expenditure of overground running was, on average, 9.6% and 8.3% higher than that of treadmill running at speeds of 8 and 9 km/h, respectively (9.36 ± 1.40 vs. 8.54 ± 1.21 kcal/min; 10.33 ± 1.55 vs. 9.54 ± 1.36 kcal/min, both p < 0.01). It is not very clear why higher energy expenditure was observed in overground running in comparison to that of treadmill running. One possible explanation is that the running mechanics of overground running is different from that of treadmill running. Kinematic and kinetic characteristics were reported between the two types; speed and contact style can affect the kinematics and kinetics of running^38,39^. The kinematics and kinetics of running were also influenced by the shoes of the subjects, which varied in style and condition^40,41^. It was shown that increasing and decreasing stride length and frequency resulted in increased metabolic cost^42–44^, and energy expenditure is lower at freely chosen stride frequency compared to running with other stride frequencies^45^. In our study, overground energy expenditure was probably increased since it might not have been the freely chosen stride frequency due to the turning of overground running and the speed control. In addition, one important possible factor was the surface-stiffness difference between the overground track and the treadmill, which affected running mechanics and induced the energy-cost difference. Several studies showed that stiffer surfaces need more aerobic demand^46–48^. We speculated that the surface of our treadmill was softer than the overground surface. Besides surface-stiffness differences, during treadmill running, the surface moves automatically, while an individual propels himself over the surface during overground running^49^. Several studies suggested that the lack of air resistance is the main reason for the difference in oxygen uptake between overground and treadmill running^6,7^. Our results showed that there was no significant difference between the energy consumption of overground and treadmill running at the speed of 7 km/h; however, the difference in energy consumption increased as speed increased. The increased differences in energy consumption may be attributed to the effect of air resistance, which becomes more pronounced as running speed increases. Energy-expenditure differences between treadmill and overground running have been attributed to running kinematic and kinetic characteristics, surface type, and environmental conditions. Furthermore, the reasons for the energy consumption of overground running being higher than that of treadmill running need further research.

Different exercise modes may result in different metabolic responses on the relative contributions of fat and carbohydrate oxidation. Studies showed that fat oxidation is significantly lower during cycling than running at the same relative intensity^14,50^. Our results found that there were significant differences in substrate metabolism and heart rate in the two modes; overground running required the runner to utilize considerably more carbohydrates but less fat than treadmill running did. CHO oxidation of overground running was, on average, 27.9% and 17.3% higher than that of treadmill running, whereas fat oxidation was, on average, 25.9% and 16% lower during overground running when compared with treadmill running at speeds of 8 and 9 km/h. These findings were consistent with a study by Kerdok^51^. Results demonstrated that overground running at speeds of 8 and 9 km/h had a higher stress response on the body than treadmill running did. Substrate metabolism is regulated by the intensity of physical activity, and we found that, the greater the activity intensity was, whatever the type of running, the larger the contribution of CHO oxidation to total energy expenditure would be, which was entirely consistent with results of previous studies^13,52^. Therefore, we should pay attention to the CHO and fat-oxidation characteristics of overground running in comparison to treadmill running to prescribe for middle-aged individuals according to the exercise aims.

Treadmill running, which is within a limited and controlled space, offers greater control compared to overground running, and is widely used by clinicians, athletes, and general population, making it close to overground running for the diagnosis and rehabilitation of injuries, training, and improving body fitness. This study suggested that overground running requires greater effort than treadmill running does as speed increases. When middle-aged women select slower running speeds than 7 km/h, they can choose overground or treadmill running according to their familiarity and comfort habits. To achieve greater energy consumption, middle-aged women are recommended to select overground over treadmill running at speeds of 8 and 9 km/h or above. If participants choose treadmill running to achieve the same physiological effects, they could increase the treadmill speed or use a 1% treadmill gradient^53^.

## Limitations and Perspectives

The study has some limitations. First, there were no measurements of sports biomechanics, such as electromyography, step, stride, and joint angular kinematics. Biomechanical data from running can provide more information on muscle activation and kinematics on overground and treadmill running. Second, substrate oxidation rates were affected by energy balance and macronutrient diet composition. Diet conditions were not monitored during the day before the test. Third, to prevent daily changes in physiological response, all exercise tests were performed on the same day, which could potentially lead to more or less fatigue for middle-aged women. Finally, the effect of VO_2max_ on metabolic parameters was not analyzed because the VO_2max_ of subjects was not tested in our study. However, several studies indicated that oxygen uptake and metabolic response during different exercises depend on an individual’s maximal aerobic power^54^. In future studies, VO_2max_ will be tested and used as a factor to analyze its influence on metabolic variables.

## Conclusion

In conclusion, VO_2_, HR, energy expenditure, and carbohydrate oxidation increased with increasing running speed in all running types. As speed increased, energy expenditure and carbohydrate oxidation were markedly higher during overground running than during treadmill running. Any differences between treadmill and overground running may lead to the incorrect prescription of physical-activity intensities. It is critical that these differences are taken into account when prescribing training intensities and choosing training modes for middle-aged women.

## Data Availability

The original data included in this study during the current study is available from the corresponding author on reasonable request.
